# Comparative Transcriptomics Reveals Key Gene Expression Differences between Diapausing and Non-Diapausing Adults of *Culex pipiens*

**DOI:** 10.1371/journal.pone.0154892

**Published:** 2016-04-29

**Authors:** David S. Kang, Michael A. Cotten, David L. Denlinger, Cheolho Sim

**Affiliations:** 1 Department of Biology, Baylor University, Waco, TX, 76798, United States of America; 2 Department of Evolution, Ecology, and Organismal Biology and Department of Entomology, Ohio State University, 318 West 12th Avenue, Columbus, OH, 43210, United States of America; Johns Hopkins University, Bloomberg School of Public Health, UNITED STATES

## Abstract

Diapause is a critical eco-physiological adaptation for winter survival in the West Nile Virus vector, *Culex pipiens*, but little is known about the molecular mechanisms that distinguish diapause from non-diapause in this important mosquito species. We used Illumina RNA-seq to simultaneously identify and quantify relative transcript levels in diapausing and non-diapausing adult females. Among 65,623,095 read pairs, we identified 41 genes with significantly different transcript abundances between these two groups. Transcriptome divergences between these two phenotypes include genes related to juvenile hormone synthesis, anaerobic metabolism, innate immunity and cold tolerance.

## Introduction

As a vector of West Nile virus and other human pathogens, the mosquito *Culex pipiens* is of growing concern in the US [1–4]. Members of the *Culex pipiens* complex of mosquitoes are virtually indistinguishable by simple morphometrics, yet they exhibit a robust range of life strategies driven by genetic architecture [5, 6]. Among members of this complex, only *Culex pipiens* form pipiens undergoes an overwintering diapause, a feature that is critical for expanding its habitat to temperate regions.

Diapause is an alternative developmental program in which the mosquito senses impending changes in its environment and adapts accordingly by entering a dormant state [7, 8]. Diapause is an anticipated response triggered by shortened day lengths and low temperature, which in turn restrict insulin signal and consequently halt the release of the isoprenoid juvenile hormone [9, 10]. This absence of juvenile hormone induces a phenotype with diverse physiological, developmental and behavioral traits including the seeking of protected overwintering sites, delayed reproductive development, stress tolerance, sugar gluttony and nutrient rationing. The adult diapause of *Cx*.*pipiens* is initiated only in females, and the programming begins during the larval and pupal stages. Post-eclosion these females will mate, but will not engage in hematophagous (blood feeding) behavior or vitellogenesis (egg yolk deposition) until termination of the diapause program [8]. These responses allow the mosquito to prepare for winter, conserve energy reserves and avoid adverse conditions. Diapause is thus a critical adaptation for survival of these vectors of human and animal disease. Despite the crucial role of diapause to mosquito survival, we know little about how mosquitoes are able translate complex environmental signals into the developmental switch that evokes the complex hormonal and physiological traits that comprise the diapause syndrome [10–18].

Diapause is a quantitative trait in which multiple genes share complex interactions that generate the phenotype. Genome wide interactions underlying this trait have been previously investigated in a variety of organisms including bumble bees, crickets, spider mites, flesh flies, apple maggots, moths, house flies and other mosquitoes [19–27]. In *Cx*. *pipiens*, several minor QTLs and a major QTL have been identified, but mapping studies have been impeded by a lack of markers [28]. Here we use RNA-seq to simultaneously quantify and identify transcriptional profiles of diapausing and non-diapausing females of *Cx*. *pipiens* to generate hypotheses that may explain the dramatic differences in these two phenotypes.

## Results

### Data Analysis

HiSeq 2000 sequencing yielded 42,175,155 total read pairs for *Cx*. *pipiens* diapausing samples, with an average read length of 101 base pairs and 8,519,373,230 total bases read. By comparison, *Cx*. *pipiens* non-diapausing samples yielded 33,447,940 total read pairs with an average read length of 101 base pairs and 6,756,483,880 total bases read. A student t-test reveals that the numbers of reads between the diapausing and non-diapausing samples were significantly different (p = 0.0132).

Approximately 56% of the diapause transcript reads uniquely aligned to the reference *Cx*. *quinquefasciatus* genome from vectorbase.org, while 54% of the non-diapausing reads aligned uniquely to the reference genome. TopHat revealed that 0.75% of the diapause reads and 0.40% of the non-diapause reads had multiple mapping sites or were of low quality. Due to the non-specific nature of these transcripts they were suppressed.

### Differential Expression

Examination of whole bodies of female adults revealed a high homology of transcripts between *Cx*. *quinquefasciatus* and *Cx*. *pipiens* in both diapausing and non-diapausing samples. The transcriptome of diapausing females contained only 4,303 unique reads out of 42,175,115 total reads when compared to the *Cx*. *quinquefasciatus* reference genome, and the non-diapausing sample expressed 4,007 unique reads out of 33,447,940 total reads. Additionally, transcripts from diapausing females of *Cx*. *pipiens* revealed 21,146 alternative splices compared to the reference *Cx*. *quinquefasciatus* genome, yielding 9,388 putative novel isoforms. Similarly, non-diapausing females revealed 20,468 alternative splices, yielding 9,142 novel isoforms.

Cufflinks Analysis of mapped reads to the reference genome was used to calculate differences in transcript abundance, expressed as FPKM. An examination of our data reveals that diapausing females of *Cx*. *pipiens* exhibited 11,193 transcripts with FPKMs below 10 (FPKM<10), 6,174 transcripts with FPKMs greater than or equal to 10 and less than 100 (10 ≤ FPKM < 100), 838 transcripts with FPKM greater than or equal to 100 and less than 1000 (100 ≤ FPKM <1000), and 129 transcripts with a FPKM greater than or equal to 1,000 (FPKM ≤1000). In comparison, the non-diapausing females of *Cx*. *pipiens* exhibited 11,061 transcripts with FPKMs below 10 (FPKM<10), 6,198 transcripts with FPKMs greater than or equal to 10 and less than 100 (10 ≤ FPKM < 100), 708 transcripts with FPKM greater than or equal to 100 and less than 1000 (100 ≤ FPKM <1000), and 259 transcripts with a FPKM greater than or equal to 1,000 (FPKM ≤1000).

**Fig 1:**
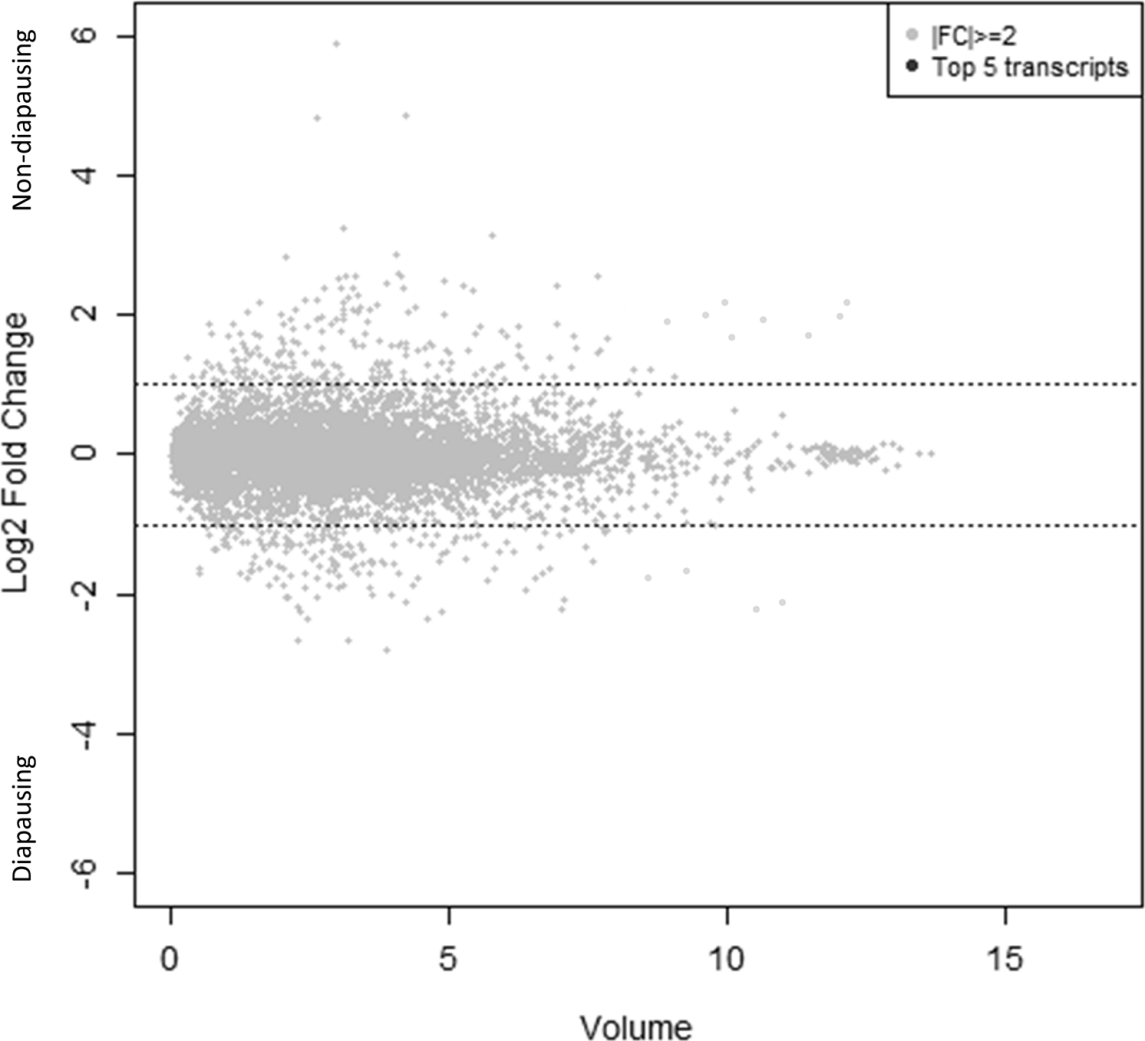
Volume plot distribution of transcripts.

**Fig 2:**
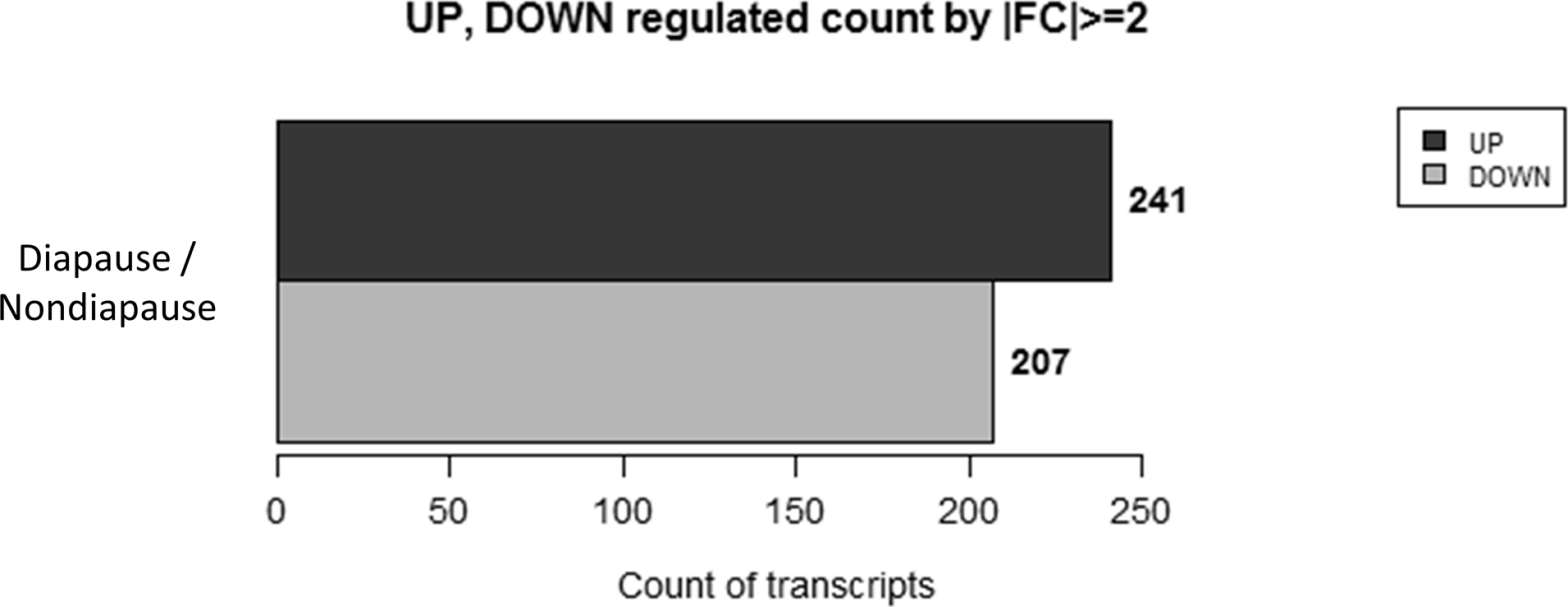
Total genes upregulated and downregulated in diapausing females of *Cx*. *pipiens*.

**Fig 3:**
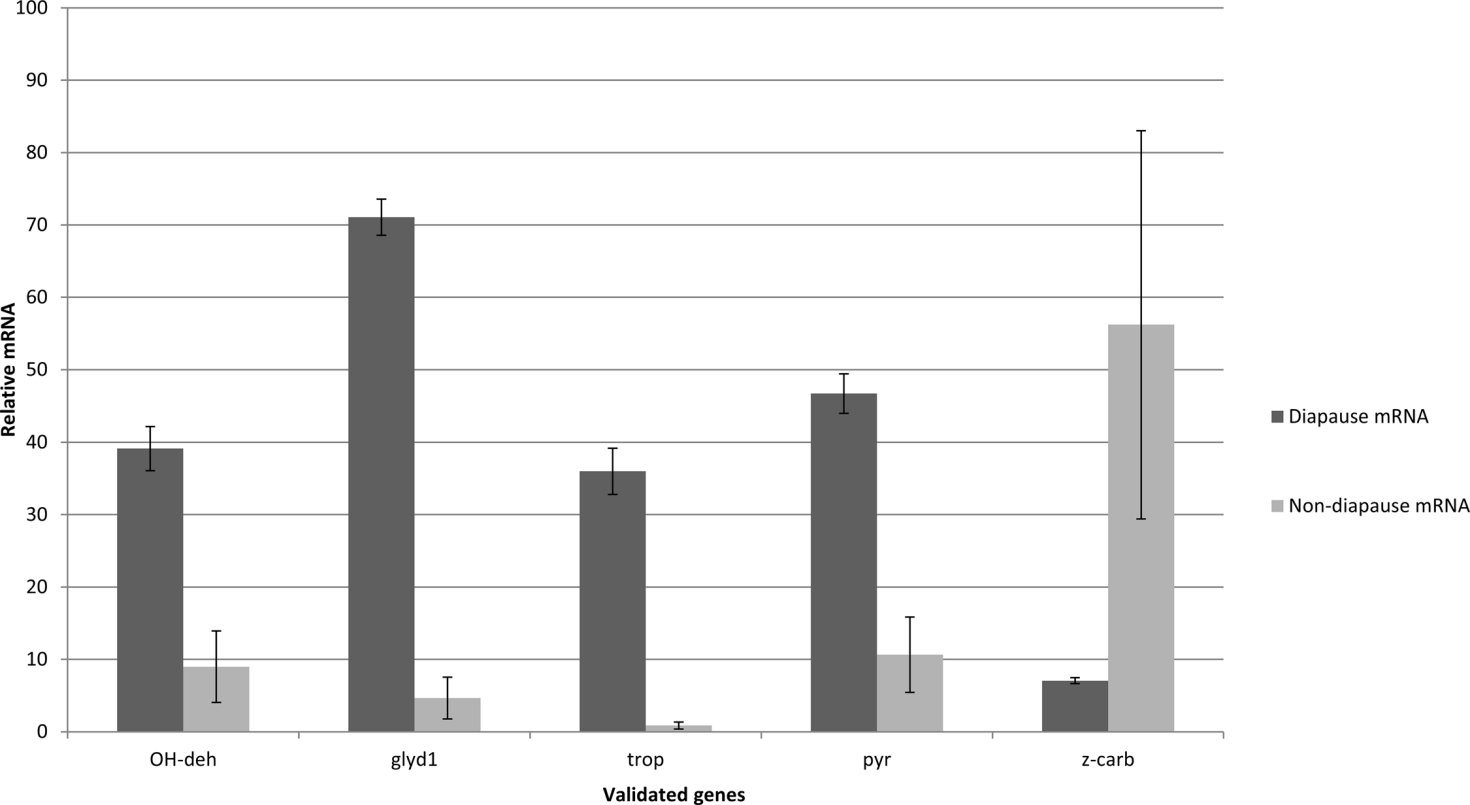
Expression abundance of diapausing vs. non-diapausing females of *Cx*. *pipiens* at 7 days after adult eclosion via quantitative real-time PCR. Ribosomal protein large subunit 19 (RpL19) as a loading control. Error bars represent standard error, n = 3.

Distribution of transcripts can be seen in a volume plot (Fig 1). Further examination of fold change differences (log_2_) revealed 241 transcripts upregulated in diapausing females and 207 transcripts downregulated in diapausing females (Fig 2). qRT-PCR validation of alcohol dehydrogenase, a glycogen debranching enzyme, troponin C, pyrroline-5-carboxylate reductase, and z-carboxypeptidase A1 precursor support the accuracy of our RNA-seq results (Fig 3).

**Fig 4:**
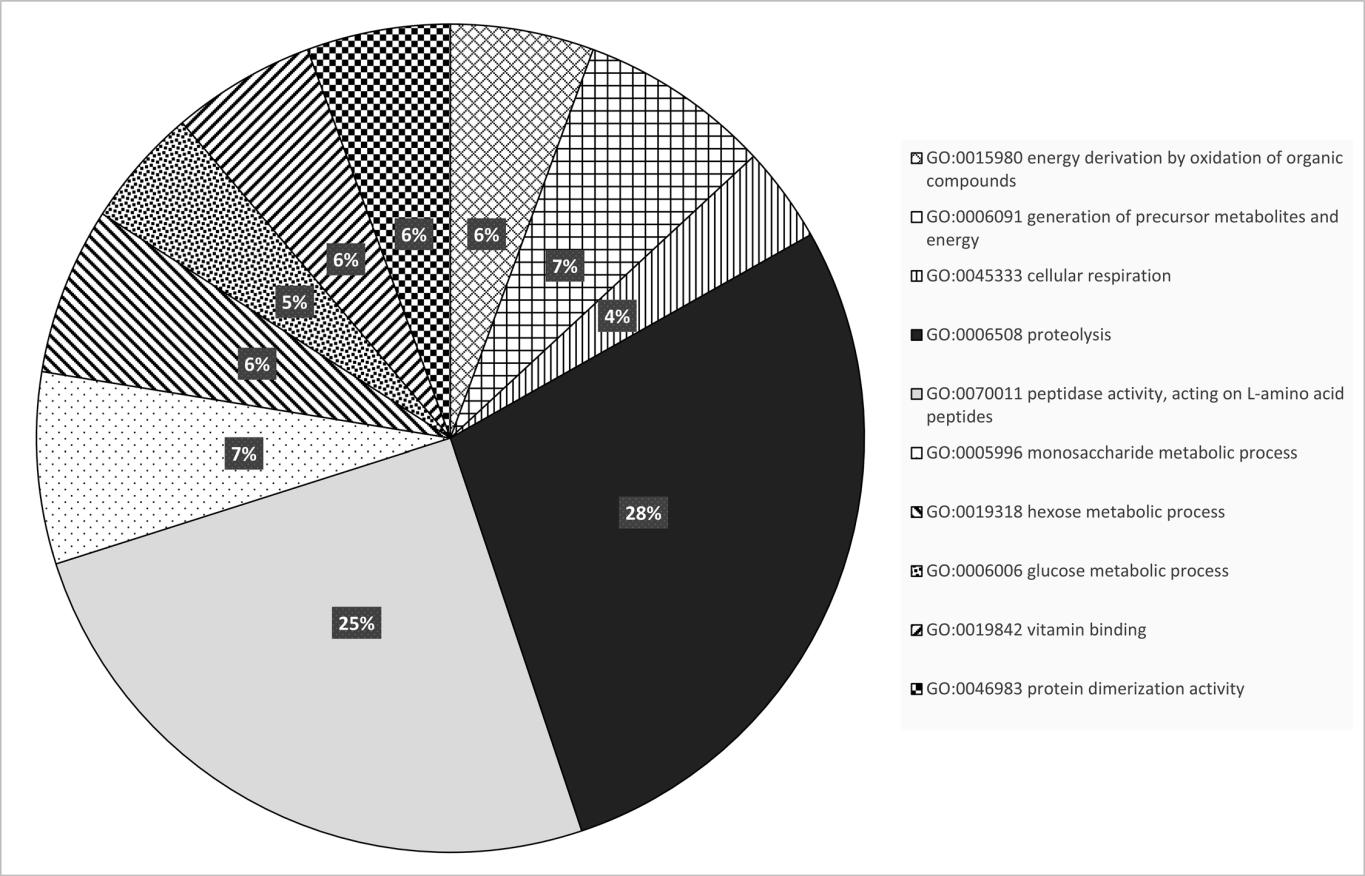
Significantly differentially expressed transcripts classified by ontology. Transcripts with significant upregulation or downregulation during diapause.

Based on P values, 41 transcripts showed a significantly different abundance between diapausing and non-diapausing females (Table 1). DAVID Analysis of the expressed transcripts that were significantly different revealed 12 genes categorized under biological process, 6 genes under cellular components and 17 genes under molecular function, with many transcripts related to glycolysis. Among these significantly different transcripts, three transcripts were mapped to known metabolic/signaling KEGG pathways involved with “starch and sucrose metabolism.” However, fourteen transcripts did not map to the reference genome, and 9 were conserved hypothetical proteins. Interestingly, diapausing females had a lower number (10) of downregulated transcripts at this threshold compared to upregulated transcripts. Gene function categories of the divergent transcripts are shown in Fig 4.

**Table 1:** Differential gene expression profiles of diapausing and non-diapausing females of *Cx*. *pipiens* 7 days after adult eclosion, using the Illumina HiSeq 2000 platform.

Gene	Locus	Description	Diapause FPKM*	Non-diapause FPKM	Log_2_ Fold Change (D/ND)
CPIJ018863	supercont3.1358:78853–81474	hyalurononglucosaminidase precursor	1.06	0	N/A
CPIJ007201	supercont3.163:705539–707969	serine protease	13.35	0	N/A
CPIJ014348	supercont3.599:237687–239756	sodium/hydrogen exchanger 8	1.325	0	N/A
CPIJ005208	supercont3.108:113365–120827	alpha-amylase	10.17	1.80	-2.50
CPIJ015401	supercont3.675:30064–44889	galactose-specific C-type lectin, putative	101.15	18.29	-2.47
CPIJ000449	supercont3.5:167316–168492	galactose-specific C-type lectin, putative	61.53	11.57	-2.41
CPIJ007618	supercont3.148:25822–56252	alcohol dehydrogenase	4.72	1.16	-2.02
CPIJ012704	supercont3.404:144065–145118	pyrroline-5-carboxylate reductase	75.81	19.86	-1.93
CPIJ005941	supercont3.104:135706–144687	ADP,ATP carrier protein 2	1297.29	384.94	-1.75
CPIJ020026	supercont3.2812:2034–6343	glycogen debranching enzyme	80.24	27.86	-1.53
CPIJ013040	supercont3.447:1311–4950	glycogen debranching enzyme	64.44	22.82	-1.50
CPIJ012251	supercont3.398:300000–334450	troponin C	769.78	275.20	-1.48
CPIJ002089	supercont3.21:536022–537672	salivary protein	113.21	45.57	-1.31
CPIJ019028	supercont3.1589:59519–69546	ran	28.75	86.02	1.58
CPIJ011998	supercont3.346:205841–207234	zinc carboxypeptidase A 1 precursor	73.26	226.73	1.63
CPIJ011172	supercont3.299:36590–73789	dynein beta chain	1.24	4.24	1.77
CPIJ020177	supercont3.2736:532–9439	nascent polypeptide associated complex alpha subunit	17.98	81.19	2.17

*RNA-seq results expressed in terms of fragments per kilobase of exon per million fragments mapped (FPKM).

**Fig 5:**
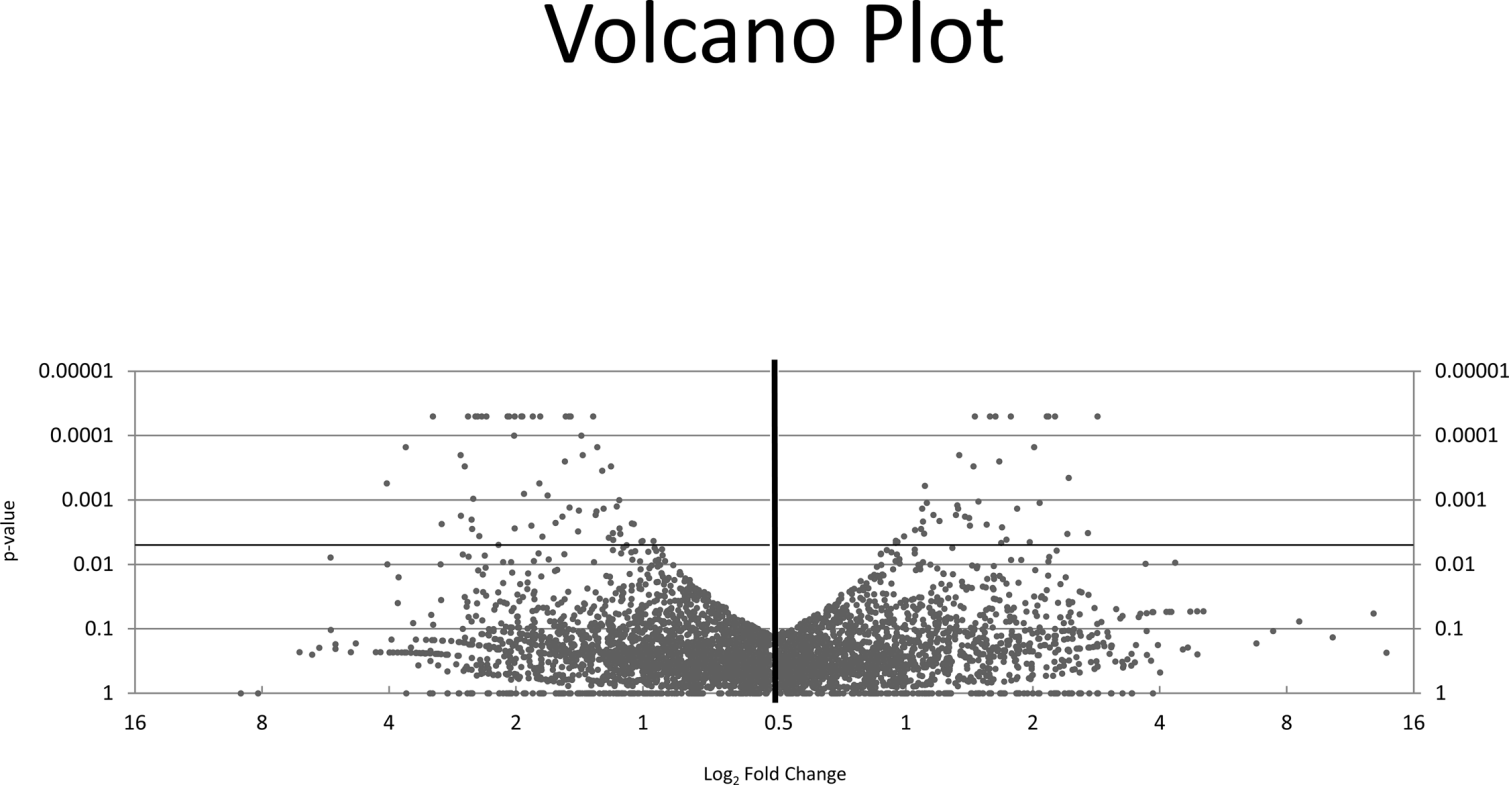
Volcano plot of log_2_ fold change vs statistical significance.

Because the number of significantly different transcripts was relatively low in the DAVID Analysis, which uses a broad genome-wide technique that focuses on categories of genes rather than individual transcripts, the ontology of each individual transcript was also manually investigated. Ontologies of individual genes were classified as relating to the juvenile hormone pathway, anaerobic metabolism, innate immunity, cold tolerance, or as hypothetical proteins (Fig 4). While the majority of upregulated transcripts were classified as hypothetical proteins, diapausing females exhibited an increase in genes related to metabolism, juvenile hormone, and cold resistance, while genes involved with metabolism and structural fortification where downregulated. Transcripts were assessed based on fold change differences, significance and ontologies for validation via volcano plot (Fig 5).

## Discussion

The increasing prevalence of West Nile Virus in the US emphasizes the need to study the molecular regulation of a key adaptation such as diapause in *Culex* mosquitoes. Furthermore, the availability of the *Cx*. *quinquefasciatus* reference genome and RNA-seq technology offers an easy, cost effective method to simultaneously identify and quantify differences in gene expression arising from divergent traits such as diapause in *Cx*. *pipiens*. We utilized transcriptional profiling to identify potential gene targets for further functional analysis in working toward our goal of understanding the basis for diapause in this vector species.

A shut-down in the production of juvenile hormone (JH) by the *corpora allata* is central to the diapause program of *Cx*. *pipiens* [29]. Application of juvenile hormone (JH) will terminate diapause in this species (21), and surgical removal of the *corpora allata* from non-diapausing females results in a simulated diapause, an effect that can also be ameliorated by the application of juvenile hormone [30]. While these results suggest a simple model of endocrine control, the diapause program has multifaceted downstream effects including links to insulin signaling and activation of the transcription factor Foxo which complicate the understanding of this dynamic suite of traits that comprise the diapause phenotype [31]. The transcript profile we present here for *Cx*. *pipiens* offers potential links to the diapause syndrome.

Allatostatin halts juvenile hormone production in *Cx*. *pipiens* and is well documented as a regulator of diapause in other insects as well [9, 32, 33]. Studies with the cockroach, *Diploptera punctata*, demonstrated that allatostatin is associated with a dose-dependent upregulation of digestive enzymes in the insect midgut with allatostatin-reactive cells transversing the midgut and basal lamina [34, 35]. As a principal carbohydrate-metabolizing enzyme in insects, alpha-amylase is specifically responsible for converting starch into maltose in *D*. *punctata* and is upregulated in the presence of allatostatin [34, 36]. Though we have no evidence that allatostatin is upregulated during diapause in *Cx*. *pipiens*, alpha-amylase is upregulated in diapausing females of *Cx*. *pipiens*, suggesting a potential link between alpha-amylase and allatostatin, and further suggesting that this digestive enzyme is associated with increased efficiency of carbohydrate uptake in diapausing females [37].

Differential gene expression analysis further revealed an increase of transcripts involved with glycolytic metabolism in diapausing females. In particular, two separately upregulated glycogen debranching enzymes suggest an increase in glycolysis and gluconeogenesis in diapausing adults. As diapausing females of *Cx*. *pipiens* are subjected to periods of fasting, starvation and low energy diets, these anticipatory preparations are unsurprising. Increased anaerobic metabolism is a trait found during dormancy of several other organisms, including *Sarcophaga crassipalpis*, *Drosophila melanogaster*, *Caenorhabditis elegans* and *Wyeomyia smithii* [19, 38–41]. When diapause is terminated in insects such as the pupal apple maggot fly, *Rhagoletis pomonella*, cessation of diapause results in an increase of metabolism to levels exhibited in non-diapausing pupae [20].

The innate immune system is a component of insect diapause that is not yet well understood, although genes associated with the immune response have been shown to be upregulated in numerous diapausing insects [42]. A transcript associated with innate immunity, serine protease, was upregulated in diapausing females of *Cx*. *pipiens*. Among their diverse roles, serine proteases are involved in hemolymph coagulation, synthesis of antimicrobial peptides and melanin, and are responsible for activation of the immune system in the presence of pathogens [43–45]. Two galactose-specific C-type lectins, were also upregulated in diapausing females. These results correspond to observations in the cotton bollworm, *Helicoverpa armigera*, a species in which the innate immune system is fortified against bacterial and fungal infections during diapause [42, 46]. These cold-tolerant calcium-dependent carbohydrate-binding pattern recognition proteins are able to recognize pathogens and serve as initiators of innate immune responses such as phagocytosis, prophenoloxidase activation and hemocyte nodule formation. [47, 48]. While serine proteases and C-type lectins are also associated with blood feeding in insects, it is unlikely that the upregulation of these enzymes and a third salivary protein in diapausing females is related to anticoagulation because none of the mosquitoes used in our experiments were offered a blood meal [45, 49, 50].

Cold tolerance is a hallmark of the diapause phenotype, as many of the physiological changes linked to diapause are associated with preparation for winter. In addition to the previously mentioned lectins, another cold tolerance gene, pyrroline-5-carboxylate reductase, has been linked with increased cold-shock tolerance in *D*. *melanogaster* and is responsible for the final step in the biosynthesis of proline [51, 52]. Increased cold tolerance in insects is commonly correlated with upregulation of proline, a potentially important source of metabolic fuel for overwintering [53–55]. The upregulation of pyrroline-5-carboxylate reductase and a hyalurononglucosaminase precursor indicate an anticipatory preparation for overwintering in diapausing *Cx*. *pipiens*. Furthermore, the upregulation of troponin C suggests fortification of structural components in diapausing individuals. In soldier termites, the presence of troponin C is associated with thickening of the cuticle and musculature, which may in turn lead to increased cold tolerance and desiccation resistance in diapausing mosquitoes [56, 57].

In contrast, diapausing females exhibited downregulation of zinc carboxypeptidase A 1 precursor, a transcript upregulated by the insect steroid hormone 20-hydroxyecdysone (48), a hormone not only involved in molting but also in coordinating reproductive processes, a feature that would be restricted to non-diapausing females of *Cx*. *pipiens* that are preparing to take a blood meal and initiate ovarian development.

While many of these ontologies offer insights into the control of diapause, the ubiquitous nature and broad categorizations of several ontological categorizations preclude speculation about the function of several candidate genes. Thus, expression differences of ran, sodium/hydrogen exchanger 8, and dynein beta chain, and a nascent polypeptide associated complex subunit have not been addressed in this manuscript but warrant future investigation because they do indeed represent major diapause/nondiapause distinctions. Certain other distinctions were not expected. For example, the upregulation of alcohol dehydrogenase in diapausing females was not anticipated because this class of enzyme has been tied to JH production and we know that JH synthesis is shut down during diapause in *Cx*. *pipiens*, but we recognize that there are numerous forms of alcohol dehydrogenases, and we cannot speculate on the nature of this specific transcript [58].

In the pitcher-plant mosquito, *Wyeomyia smithii*, the instar at which photoperiodic initiation of diapause occurs is correlated with latitude. Despite this conspecific variability within *W*. *smithii* biotypes the circadian genes controlling the photoperiodic induction of diapause are conserved across populations [21, 22, 59, 60]. As both diapausing and non-diapausing *Cx*. *pipiens* mosquitoes shared an identical genetic ancestry, it is unlikely that differences between the two programs are due to sequence variation. Thus, it is interesting that our study did not find significant differences in expression of the clock genes between diapausing and nondiapausing *Cx*. *pipiens*. A previous study with *Cx*. *pipiens* has shown that expression of the clock genes is diminished in later stages of adult diapause in this species, thus we anticipate that such differences would be apparent if we had compared different phases of diapause or prediapause development [61–63].

Fragmentation of the *Cx*. *quinquefasciatus* genome (N50 = 476 kb) and low chromosomal assignment of the total genome assembly (13%) emphasize the need for further investigation of the *Cx*. *pipiens* complex [64, 65]. Furthermore, the absence of a full physical map limits the prospect of genome-wide association studies, and many transcripts are hypothetical proteins of unknown ontology. Fortunately, the ontologies of many of our transcripts correspond with previous studies of diapause and validate the accuracy of our results, but one of the most exciting aspect of our results are the nine hypothetical proteins and the unknown transcripts associated with the diapause syndrome; revealing their identities and roles will likely be critical for understanding the important suite of adaptations that comprise the diapause phenotype (Table 2).

**Table 2:** Hypothetical proteins and their positions on the *Culex quinquefasciatus* (Johannesburg strain) reference genome.

Gene ID	Locus	Diapause FPKM*	Non-diapause FPKM	Log_2_ Fold Change (D/ND)
CPIJ006495	supercont3.125:61017–61995	3.38626	24.333	-2.84515
CPIJ011623	supercont3.329:415108–415963	10.1893	48.7056	-2.25703
CPIJ012164	supercont3.368:82667–83532	37.5804	116.199	-1.62854
CPIJ012185, CPIJ012186	supercont3.374:183520–184852	50.4615	138.54	-1.45704
CPIJ013706	supercont3.514:131855–159297	169.444	64.1957	1.40026
CPIJ014352	supercont3.599:260569–262212	17.4696	1.97028	3.14837
CPIJ015860	supercont3.679:55208–56318	80.7776	20.9854	1.94457
CPIJ016534	supercont3.772:2934–4658	116.778	27.6686	2.07745
CPIJ016689	supercont3.792:100918–188468	637.201	105.029	2.60096

*RNA-seq results expressed in terms of fragments per kilobase of exon per million fragments mapped (FPKM).

The fact that this study examines only a single time point during diapause obviously reduces the richness that we suspect would be evident if the full course of diapause were to be examined. It is our hope that a more comprehensive investigation of the functional roles of the genes described in this study, along with an expansion to additional time points, will result in a clearer understanding of the intriguing diapause phenotype.

## Materials and Methods

### Mosquito Rearing

The colony of *Culex pipiens* originated from wild-caught mosquitoes collected in Columbus, Ohio in 2000 (35) and maintained at Baylor University since 2010. As previously described, non-diapausing adults were generated using a 15 hour light: 9 hour dark (L:D) daily light cycle at 18°C and 75% relative humidity. Diapausing adults were reared under a 9:15 L:D cycle at 18°C and 75% relative humidity [44]. Diapause was confirmed by measurement of the primary ovarian follicles and germaria as previously described [66]. Larvae were reared in de-chlorinated tap water and fed Tetramin fish food (Tetra Holding Inc., Blacksburg, VA). Adults were maintained on honey-soaked sponges and kept in large screened cages.

### Total RNA Extraction

Total RNA was extracted from two sample sets that are reared either from diapausing (short daylength) or non-diapausing conditions (long daylength). Each biological replicate was collected from three batches of 15 adult female mosquitoes 7 days after adult eclosion using TRIzol (Life Technologies, Carlsbad, CA). Total RNA purity was tested using a NanoDrop spectrophotometer (NanoDrop Technologies, Wilmington, DE). Biological replicates were pooled for library preparation and sequencing.

### Library Preparation and Sequencing

Samples were then used for TruSeq mRNA library construction. Samples were purified twice using poly-T oligo-attached magnetic beads, before fragmentation and priming for cDNA synthesis. cDNA was synthesized using reverse transcriptase and random primers adapted into double stranded (ds) cDNA, which was then removed with Ampure XP beads (Beckman Coulter, Pasadena, CA). ds cDNA was end repaired, converting any resulting overhangs into blunt ends, before adapter adenylation of the 3’ end for pair-ended ligation. Next, adapters were ligated to ds cDNA, which was selectively amplified by PCR.

After quality control, bridge amplification was performed on a flow cell, which was loaded into a HiSeq 2000 Illumina platform. A single molecular array was synthesized with reverse termination, resulting in unique clusters of nucleotides strands which were loaded for extension and imaging. Resulting clusters were extended one base at a time with nucleotides containing reversible fluorophores, resulting in clusters that gave a single, unified signal for each base.

### Data Analysis

Reads were aligned using TopHat v1.3.3 against the *Culex quinquefasciatus* Johannesburg strain reference genome as found on VectorBase (https://www.vectorbase.org). TopHat employs the short read aligner Bowtie to identify exon splice junctions [67]. Next, Cufflinks (v2.0.2) was used to assemble transcripts, estimate abundance and test for differences in RNA expression. Additionally, Cufflinks identifies alternative isoforms of target genes, as it does not use existing genetic annotations [68]. Cufflinks then extrapolates relative transcript abundance from normalized reads and expresses the results in Fragments per Kilobase of exon per Million fragments mapped (FPKM), where FPKM is calculated as 10^9^ X number of mappable exon reads / (number of total mappable reads X number of base pairs in the exon). Cuffdiff was used to highlight significant differences in transcript expression, splicing, and promoter usage. FPKM values of diapausing and non-diapausing *Cx*. *pipiens* were comparatively examined and expressed in log_2_, where gene targets downregulated in diapausing cohorts exhibited positive fold changes, while targets up regulated in diapausing cohorts expressed negative fold changes. Transcripts were visualized to compare FPKM significance on a volcano plot. Transcripts with significant fold changes were screened for relevant ontologies.

### Gene Ontology

DAVID (Database for Annotation, Visualization and Integrated Discovery) bioinformatics resource v 6.7 was utilized to cluster significant changes in gene expression [69]. Genes were classified by biological process, cellular component and molecular function via the GO (Gene Ontology) and KEGG (Kyoto Encyclopedia of Genes and Genomes) databases.

### qRT-PCR Validation

Transcript abundances of genes with known ontologies were screened to identify candidates of interest. Next, qRT-PCR validation was performed on five candidate genes associated with different functions using an iQ5 real-time PCR detection system (Bio-Rad, Hercules, CA). 50 ng DNA was reverse transcribed and amplified via superscript III RNase H-reverse transcriptase (Invitrogen, Carlsbad, CA), per the manufacturer’s protocol, and compared to ribosomal protein L19 (RpL19), an endogenous housekeeping gene, as an internal control. Transcript divergence from the qRT-PCR results was evaluated for statistical significance using the Student’s t-test. Candidate genes and primer information are reported in Table 3.

**Table 3:** qRT-PCR primers and associated genes.

GeneID	Primer IDs	Primers	
CPIJ007618	alcohol dehydrogenase	Forward	CTGTTGGAAGCTGGAGGAGA
	(OH-deh)	Reverse	CTCTCACGTACACCATTGCG
CPIJ020026	glyocogen debranching enzyme	Forward	CATGTACAAGGACACGCTCG
	(glyd1)	Reverse	GGAGTTGTCGTAGTTTCCGC
CPIJ012251	troponin C	Forward	GACAAGACCGGCCACATTC
	(trop)	Reverse	CATCATGAAGACCTCGCGC
CPIJ012704	pyrroline-5-carboxylate reductase	Forward	AGGCAAGCTGTTCATTTCGG
	(pyr)	Reverse	TTCCAACCGATTCGAACAGC
CPIJ011998	z-carboxypeptidase A1 precursor	Forward	CTGGAGAGCACACACCAAAC
	(z-carb)	Reverse	CATCCCAACTGTCATCGCTG
